# Biological aging, left ventricular dysfunction and mortality in patients with heart failure with preserved ejection fraction

**DOI:** 10.1038/s41514-025-00281-6

**Published:** 2025-12-17

**Authors:** Xinghao Xu, Zihao Huang, Xingfeng Xu, Menghui Liu, Rihua Huang, Zhenyu Xiong, Yue Guo, Shaozhao Zhang, Ziwei Zhou, Ziyue Tang, Xinxue Liao, Xiaodong Zhuang

**Affiliations:** 1 Department of Cardiology, The First Affiliated Hospital of Sun Yat-Sen University, Guangzhou, China; 2 NHC Key Laboratory of Assisted Circulation (Sun Yat-Sen University), Guangzhou, China; 3 Department of Rehabilitation Medicine, The First Affiliated Hospital of Sun Yat-Sen University, Guangzhou, China

**Keywords:** Biomarkers, Cardiovascular diseases

## Abstract

Accelerated biological aging (BA) is linked to adverse cardiovascular events, but its role in heart failure with preserved ejection fraction (HFpEF) remains unclear. We analyzed 1,727 HFpEF patients from RED-CARPET Study (ChiCTR2000039901), assessing BA using Klemera-Doubal and PhenoAge methods. During a median 4.9-year follow-up, 321 all-cause and 180 cardiovascular deaths occurred. After full adjustment, per 1-SD increase in BA acceleration showed significantly higher risk of all-cause mortality (KDMAge HR 1.55, 95% CI 1.40-1.72; PhenoAge HR 1.24, 95% CI 1.11-1.40) and cardiovascular mortality (KDMAge HR 1.47, 95% CI 1.28-1.69; PhenoAge HR 1.21, 95% CI 1.04-1.41). BA acceleration was also significantly related to increased left ventricular mass index (LVMI), relative wall thickness, and E/e’ ratio. Mediation analysis revealed that both LVMI and the E/e’ ratio partially mediated the relationship between BA acceleration and mortality. These findings suggest BA acceleration may serve as a key prognostic marker in patients with HFpEF.

## Introduction

Heart failure with preserved ejection fraction (HFpEF) is a subtype of heart failure (HF) characterized by elevated left or right ventricular filling pressures or reduced cardiac output, despite a left ventricular ejection fraction (LVEF) ≥ 50%^1–3^. Globally, over 64 million individuals are affected by HF, and HFpEF contributes to at least 50% of the population with a rising prevalence driven by an aging population^3–5^. The five-year survival for hospitalized HFpEF remains low, although it is slightly higher than that in HF reduced ejection fraction^5^. As global aging accelerates, the burden of age-related cardiovascular diseases is expected to increase further.

Aging is a complex biological process characterized by the gradual deterioration of physiological integrity at the cellular, tissue, and organ levels^6^. With advancing age, prolonged exposure to injurious stimuli (e.g., hypertension, dyslipidemia, oxidative stress, chronic low-grade inflammation)^7,8^, along with interactions among comorbidities (anemia, chronic kidney disease, diabetes), and disease modifiers (sex, genetics), as well as limited cardiac regenerative capacity and vascular aging, collectively contribute to HF phenotypes and progression^9,10^. Chronological age (CA) is a convenient measure to assess aging status; however, individuals with the same CA may experience different rates of biological decline, making CA insufficient to reflect multisystem physiological changes^7^. In contrast, various metrics of biological age (BA) have been developed to capture the multifaceted nature of aging, including telomere length^11^, DNA methylation-based epigenetic clocks^12^, and algorithms integrating multiple clinical biomarkers^13^. These biomarkers enable a more comprehensive assessment of biological aging or aging rate. While many of these methods are costly and not routinely used in clinical practice, phenotypic age (PhenoAge) and BA derived from the Klemera and Doubal method (KDMAge) are based on easily obtainable physical measurements and systemic biochemical markers^13–15^, allowing earlier, more cost-effective, and more comprehensive detection of physiological changes and been increasingly applied to estimate BA in recent years^16–18^.

Both PhenoAge and KDMAge have demonstrated robust predictive value for mortality, cardiac structure in the healthy population^19,20^. Moreover, our previous study found that increased biomarker-based BA was associated with higher all-cause mortality, cardiovascular mortality, and hospitalization rates in patients with heart failure with reduced ejection fraction, highlighting its prognostic value^21^. However, its association with cardiac morphology and its prognostic utility for HF-related outcomes in individuals with HFpEF remain understudied^19^. Therefore, this study aims to investigate the relationship between biomarker-based BA and cardiac structure, as well as its predictive value for mortality in Chinese individuals with HFpEF.

## Results

### Baseline characteristics

The baseline characteristics of 1727 HFpEF patients grouped by sex are shown in Table 1, and echocardiographic characteristics are shown in Supplementary Table 1. The mean age of the total patients was 68.21 ± 10.63 years, and 1049 (60.7%) were males. The mean H2FPEF scores of the total patients were 3.80 ± 1.72, and females had higher scores than males. Males (*n*=1049) exhibited younger age profiles, a higher proportion of drinkers, smokers, history of coronary artery disease, PCI or cardiac surgery, and chronic kidney diseases, but lower proportions of hypertension, diabetes, and atrial fibrillation compared to females. Both male and female patients with HFpEF in this cohort had mean LVMI and E/e′ values that exceeded the respective sex-specific reference ranges.

**Table 1:** Baseline characteristics of participants

	Overall (*N* = 1727)	Male (*N* = 1049)	Female (*N* = 678)	*P* value
Age	68.21 ± 10.63	66.37 ± 10.92	71.06 ± 9.49	<0.001
Drinking	246 (14.2)	236 (22.5)	10 (1.5)	<0.001
Smoking	586 (33.9)	565 (53.9)	21 (3.1)	<0.001
BMI, kg/m^2^	24.57 ± 3.72	24.50 ± 3.62	24.69 ± 3.88	0.311
SBP, mm Hg	134.74 ± 20.83	133.62 ± 20.42	136.48 ± 21.35	0.005
DBP, mm Hg	75.18 ± 12.97	75.37 ± 13.33	74.89 ± 12.41	0.449
H_2_FPEF score, points	3.80 ± 1.72	3.61 ± 1.68	4.06 ± 1.74	<0.001
**Comorbidities**				
Hypertension	1371 (79.4)	803 (76.5)	568 (83.8)	<0.001
Coronary artery disease	1395 (80.8)	873 (83.2)	522 (77.0)	0.002
PCI or cardiac surgery	267 (15.5)	187 (17.8)	80 (11.8)	0.008
Diabetes	787 (45.6)	437 (46.7)	350 (57.3)	<0.001
Atrial fibrillation	243 (14.1)	134 (12.8)	109 (16.1)	0.063
Stroke	119 (6.9)	73 (7.0)	46 (6.8)	0.966
Chronic kidney disease	177 (10.2)	123 (11.7)	54 (8.0)	0.015
**Medication**				
Lipid lowering medications	1618 (93.7)	991 (94.5)	627 (92.5)	0.118
Antihypertension medications	1690 (97.9)	1030 (98.2)	660 (97.3)	0.311
ACEi/ARB/ARNI	1379 (79.8)	814 (77.6)	565 (83.3)	0.208
Beta-blockers	1510 (87.4)	920 (87.7)	590 (87.0)	0.964
Oral hypoglycemic agents	583 (33.8)	329 (31.4)	254 (37.5)	0.010
Insulin use	433 (25.1)	254 (24.2)	179 (26.4)	0.333
SGLT2 inhibitors	192 (11.1)	124 (11.8)	68 (10.0)	0.850
**Laboratory data**				
HDL-C, mg/dL	40.44 ± 10.61	38.52 ± 9.88	43.40 ± 11.01	<0.001
LDL-C, mg/dL	101.73 ± 33.90	99.47 ± 33.88	105.23 ± 33.65	0.001
Triglyceride, mg/dL	138.34 ± 108.96	132.07 ± 109.43	148.03 ± 107.59	0.003
BUN, mg/dL	44.04 ± 23.75	44.75 ± 24.26	42.95 ± 22.92	0.125
Creatine, umol/L	109.70 ± 110.10	119.00 ± 119.13	96.25 ± 93.88	<0.001
Albumin, g/dL	38.54 ± 4.48	38.54 ± 4.49	38.53 ± 4.48	0.958
WBC count, million cells/uL	7.36 ± 2.28	7.48 ± 2.18	7.16 ± 2.41	0.004
RBC count, million cells/uL	4.37 ± 0.72	4.49 ± 0.75	4.19 ± 0.62	<0.001
NTproBNP	212.6 [78.6, 695.5]	203.8 [74.2, 630.0]	217.4 [86.0, 780.0]	0.821
**Biological Age**				
KDMAge	80.91 ± 14.59	81.12 ± 15.52	80.58 ± 13.02	0.455
KDMAge acceleration	12.69 ± 11.61	14.75 ± 11.61	9.52 ± 10.88	<0.001
PhenoAge	65.98 ± 11.32	65.41 ± 11.66	66.87 ± 10.72	0.009
PhenoAge acceleration	-2.23 ± 4.81	-0.96 ± 4.40	-4.20 ± 4.74	<0.001

Data are shown as *n* (%), mean ± SD, or median [25th–75th percentile]. Baseline characteristics of the 1727 eligible HFpEF patients from the RED-CARPET study, stratified by sex. There were significant between-group differences in most covariates considered.

*BMI* body mass index, *SBP* systolic blood pressure, *DBP* diastolic blood pressure, *PCI* percutaneous coronary intervention, *ACEi* angiotensin converting enzyme inhibitor, *ARB* angiotensin receptor blocker, *ARNI* angiotensin receptor–neprilysin inhibitor, *SGLT2i* sodium–glucose cotransporter 2 inhibitor, *HDL-C* high-density lipoprotein cholesterol, *LDL-C* low-density lipoprotein cholesterol, *BUN* blood urea nitrogen, *WBC* white blood cell, *RBC* red blood cell, *KDMAge* Klemera-Doubal method age.

Correlations between BA measures and CA were displayed in Supplementary Fig. 2. As expected, CA was strongly correlated with KDMAge (Pearson coefficient = 0.62) and PhenoAge (Pearson coefficient = 0.91). Following chronological age adjustments, residual values for KDMAge and PhenoAge (term as ‘biological age acceleration, BA acceleration’) maintained their correlation.

### Association between biological age and mortality

**Fig. 1:**
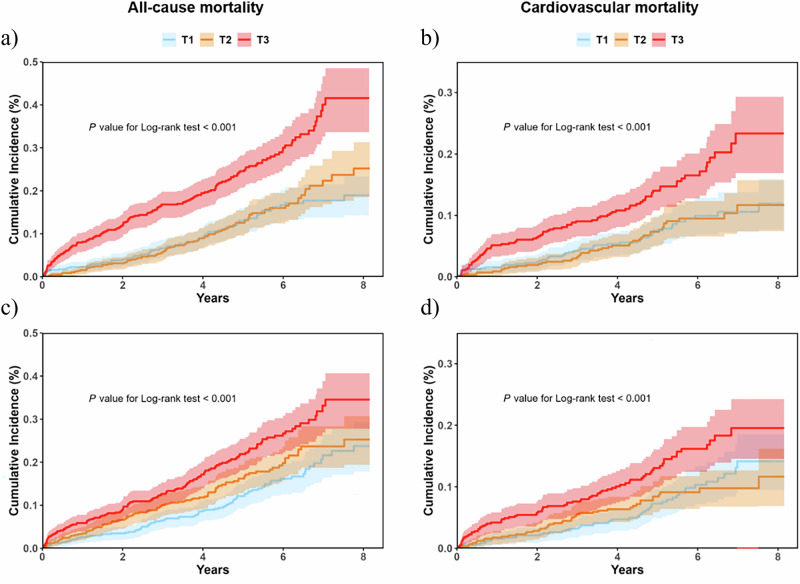
Cumulative incidence of all-cause mortality and cardiovascular mortality by tertiles of KDMAge acceleration and PhenoAge acceleration. **a** KDMAge acceleration and all-cause mortality. **b** KDMAge acceleration and cardiovascular mortality. **c** PhenoAge acceleration and all-cause mortality. **d** PhenoAge acceleration and cardiovascular mortality.

During a median follow-up of 4.9 years, 321 cases of all-cause mortality and 180 cases of cardiovascular mortality were recorded for analysis. Table 2 shows the Cox proportional hazard analysis of the association between the BA acceleration and mortality. After adjusting for all potential confounders, per 1-SD increase in BA acceleration showed significantly higher risk of all-cause mortality (KDMAge acceleration: HR 1.55, 95% CI 1.40–1.72, *P* < 0.001; PhenoAge acceleration: HR 1.24, 95% CI 1.11–1.40, *P* < 0.001) and cardiovascular mortality (KDMAge acceleration: HR 1.47, 95% CI 1.28–1.69, *P* < 0.001; PhenoAge acceleration: HR 1.21, 95% CI 1.04–1.41, *P* = 0.015). Compared with the first tertile of BA acceleration, participants in the third tertile appeared to have a highest risk of all-cause mortality (KDMAge acceleration: HR 2.31, 95% CI 1.73–3.09, *P* < 0.001; PhenoAge acceleration: HR 1.66, 95% CI 1.24–2.22, *P* < 0.001) and cardiovascular mortality (KDMAge acceleration: HR 2.06, 95% CI 1.39–3.05, *P* < 0.001; PhenoAge acceleration: HR 1.64, 95% CI 1.12–2.39, *P* = 0.009) (Table 2, Fig. 1). In sensitivity analyses additionally adjusting for NT-proBNP, the associations between BA acceleration and both all-cause and cardiovascular mortality remained significant (Supplementary Table 2).

**Table 2:** Associations of BA acceleration with all-cause mortality and cardiovascular mortality

		Model 1		Model 2		Model 3	
	Event/total (%)	HR (95% CI)	*P* value	HR (95%CI)	*P* value	HR (95%CI)	*P* value
KDMAge acceleration							
All-cause mortality							
per SD increase	321/1727 (18.6)	1.62 (1.47, 1.78)	<0.001	1.59 (1.44, 1.75)	<0.001	1.55 (1.40, 1.72)	<0.001
Tertile1	80/575 (13.9)	Reference	–	Reference	–	Reference	–
Tertile2	85/575 (14.8)	1.13 (0.83, 1.55)	0.409	1.22 (0.89, 1.67)	0.206	1.18 (0.86, 1.62)	0.287
Tertile3	156/577 (27.0)	2.41 (1.83, 3.19)	<0.001	2.40 (1.81, 3.18)	<0.001	2.31 (1.73, 3.09)	<0.001
* P* for trend		<0.001		< 0.001		< 0.001	
Cardiovascular mortality							
per SD increase	180/1727 (10.4)	1.54 (1.34, 1.77)	<0.001	1.51 (1.32, 1.73)	<0.001	1.47 (1.28, 1.69)	<0.001
Tertile1	48/575 (8.3)	Reference	–	Reference	–	Reference	–
Tertile2	45/575 (7.8)	1.03 (0.67, 1.57)	0.879	1.12 (0.73, 1.72)	0.579	1.08 (0.70, 1.66)	0.706
Tertile3	87/577 (15.1)	2.21 (1.52, 3.21)	<0.001	2.16 (1.48, 3.16)	<0.001	2.06 (1.39, 3.05)	<0.001
*P* for trend		<0.001		<0.001		<0.001	
PhenoAge acceleration							
All-cause mortality							
per SD increase	321/1727 (18.6)	1.27 (1.13, 1.42)	<0.001	1.26 (1.12, 1.41)	<0.001	1.24 (1.11, 1.40)	<0.001
Tertile1	85/575 (14.8)	Reference	–	Reference	–	Reference	–
Tertile2	100/575 (17.4)	1.20 (0.89, 1.61)	0.229	1.23 (0.91, 1.66)	0.160	1.24 (0.92, 1.68)	0.145
Tertile3	136/577 (23.6)	1.62 (1.22, 2.17)	<0.001	1.66 (1.24, 2.22)	<0.001	1.66 (1.24, 2.22)	<0.001
* P* for trend		<0.001		<0.001		<0.001	
Cardiovascular mortality							
per SD increase	180/1727 (10.4)	1.24 (1.06, 1.45)	0.006	1.23 (1.05, 1.44)	0.010	1.21 (1.04, 1.41)	0.015
Tertile1	52/575 (9.0)	Reference	–	Reference	–	Reference	–
Tertile2	46/575 (8.0)	0.96 (0.64, 1.46)	0.880	0.99 (0.65, 1.50)	0.985	0.93 (0.61, 1.40)	0.738
Tertile3	82/577 (14.2)	1.62 (1.11, 2.38)	0.012	1.66 (1.12, 2.44)	0.009	1.64 (1.12, 2.39)	0.009
* P* for trend		0.005		0.004		0.005	

*BA* biological age, *HR* hazard ratio, *CI* confidence interval, *Model 1* adjusted by sex, age, *Model 2* adjusted by model 1 + drinking, smoking, history of hypertension, coronary artery disease, diabetes, atrial fibrillation, and stroke, *Model 3* adjusted by model 2 + lipid lowering medications, antihypertension medications, oral hypoglycemic agent,s and insulin use.

**Fig. 2:**
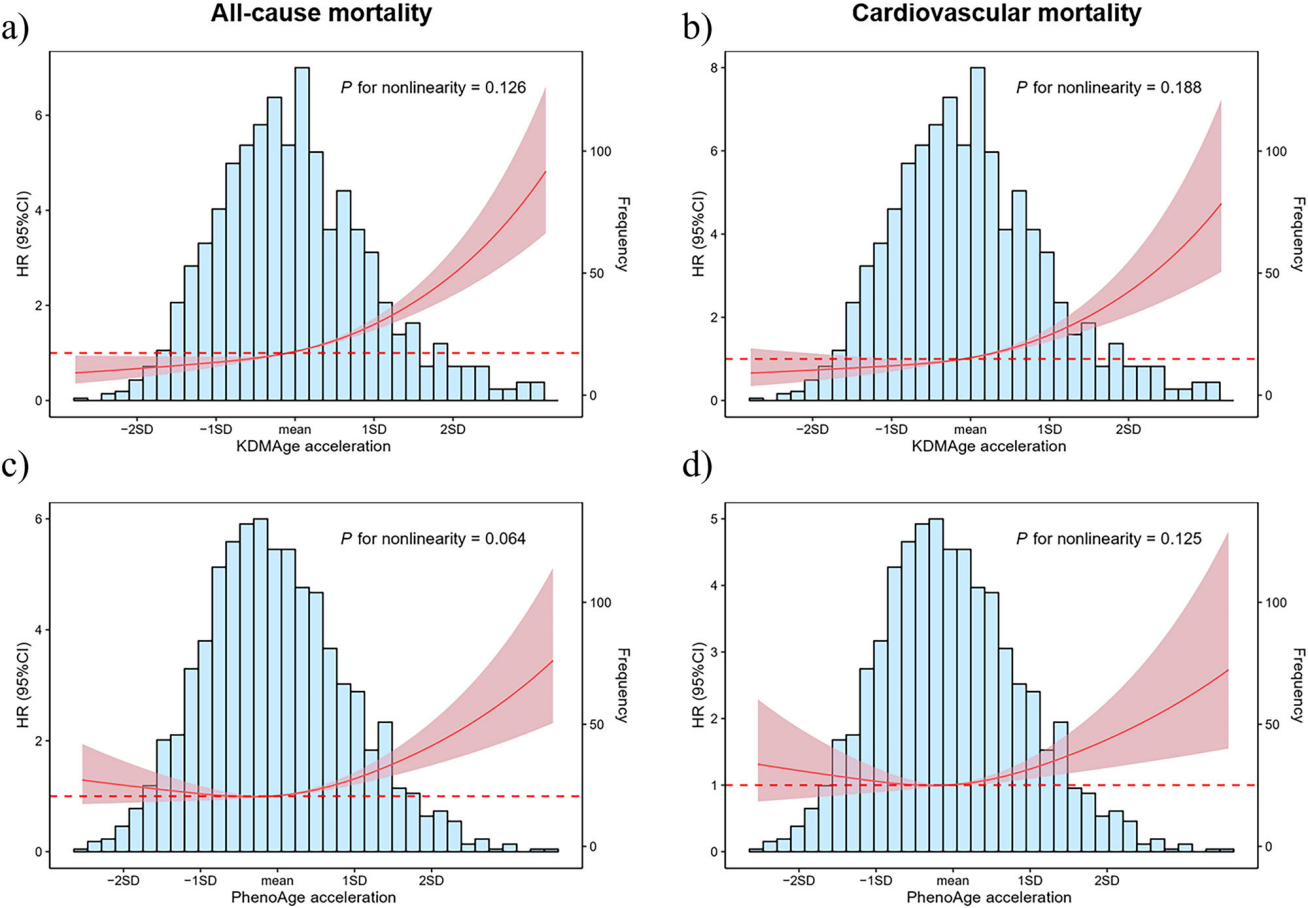
Graphs of the best-fitting models for relationships of KDMAge acceleration and PhenoAge acceleration with all-cause mortality and cardiovascular mortality. **a** KDMAge acceleration and all-cause mortality. **b** KDMAge acceleration and cardiovascular mortality. **c** PhenoAge acceleration and all-cause mortality. **d** PhenoAge acceleration and cardiovascular mortality. The blue columns represent the distribution density of the KDMAge acceleration and PhenoAge acceleration. The red dashed line represents HR = 1. The red solid line shows the HR value. The red shaded area represents the 95% CI. Restricted cubic spline regression model (3 knots) adjusted for sex, age, drinking, smoking, history of hypertension, coronary artery disease, diabetes, atrial fibrillation and stroke, use of lipid lowering medications, antihypertension medications, oral hypoglycemic agents and insulin use. HR hazard ratios, CI confidence interval.

Multivariable adjusted restricted cubic splines regression models further showed a linear association between the BA acceleration with the risk of all-cause mortality and cardiovascular mortality (Fig. 2). Results of subgroup analyses are shown in Supplementary Fig. 3. Increased KDMAge acceleration (per 1-SD) was consistently related to all-cause mortality and cardiovascular mortality (Supplementary Fig. 3a, b) in various subgroups, including sex (male or female), age (≤70 or >70 years), BMI (≤24 or >24 kg/m2), smoking (yes or no), diabetes (yes or no), hypoglycemic agents (yes or no), and hypertension (yes or no). Notably, there was significant interaction in the age subgroup (*P* for interaction = 0.002) and hypoglycemic agents subgroup (*P* for interaction = 0.008) of the association between KDMAge acceleration and all-cause mortality, with a stronger significant association in participants aged ≤70 years and those used hypoglycemic agents. The association between PhenoAge acceleration and mortality among various groups was consistent with KDMAge acceleration (Supplementary Fig. 3c, d).

### Association between biological age and LV structure and function

The relationship between BA acceleration and LV structural and functional indices are shown in Table 3. For LV structure, after covariates were fully adjusted, higher BA acceleration levels (per 1-SD) were significantly correlated with higher LVMI (KDMAge acceleration: β = 8.87, *P* < 0.001; PhenoAge acceleration: β = 3.34, *P* = 0.015) and higher RWT (KDMAge acceleration: β = 0.006, *P* = 0.010; PhenoAge acceleration: β = 0.004, *P* = 0.038). For LV diastolic function, increased BA acceleration levels were significantly correlated with higher *E*/*e*’ ratio (KDMAge acceleration: β = 0.641, *P* < 0.001; PhenoAge acceleration: β = 0.341, *P* = 0.012) in fully adjusted models. No correlation between BA acceleration and E/A and LVEF was observed.

**Table 3:** Associations of BA acceleration with left ventricular structure and function

	Model 1		Model 2		Model 3	
	β (95% CI)	*P* value	β (95% CI)	*P* value	β (95% CI)	*P* value
LV structure						
LVMI, g/m^2^						
KDMAge acceleration	9.51 (6.98, 12.05)	<0.001	9.36 (6.81, 11.91)	<0.001	8.87 (6.25, 11.50)	<0.001
PhenoAge acceleration	4.28 (1.64, 6.93)	0.001	4.11 (1.45, 6.77)	0.002	3.34 (0.62, 6.07)	0.015
RWT						
KDMAge acceleration	0.006 (0.002, 0.01)	0.005	0.006 (0.002, 0.01)	0.005	0.006 (0.001, 0.01)	0.010
PhenoAge acceleration	0.005 (0.001, 0.009)	0.020	0.005 (0.001, 0.009)	0.022	0.004 (0.0002, 0.009)	0.038
LV systolic function						
LVEF, %						
KDMAge acceleration	−0.303 (−0.663, 0.056)	0.098	−0.310 (−0.668, 0.047)	0.089	−0.176 (−0.545, 0.191)	0.347
PhenoAge acceleration	−0.272 (−0.642, 0.097)	0.148	−0.285 (−0.654, 0.083)	0.129	−0.144 (−0.521, 0.232)	0.452
LV diastolic function						
E/A						
KDMAge acceleration	0.008 (−0.017, 0.034)	0.526	0.013 (−0.011, 0.038)	0.302	−0.003 (−0.028, 0.023)	0.837
PhenoAge acceleration	0.013 (−0.014, 0.041)	0.340	0.018 (−0.008, 0.045)	0.174	0.003 (−0.024, 0.030)	0.835
*E/e*’ ratio, %						
KDMAge acceleration	0.889 (0.638, 1.141)	<0.001	0.887 (0.637, 1.138)	<0.001	0.641 (0.388, 0.894)	<0.001
PhenoAge acceleration	0.613 (0.344, 0.881)	<0.001	0.601 (0.333, 0.869)	<0.001	0.341 (0.073, 0.609)	0.012

*BA* biological age, *LVMI* left ventricular mass index, *RWT* Relative wall thickness, *HR* hazard ratio, *CI* confidence interval.

*Model 1* adjusted by sex, age, *Model 2* adjusted by model 1 + drinking, smoking, history of hypertension, coronary artery disease, diabetes, atrial fibrillation, and stroke, *Model 3* adjusted by model 2 + lipid lowering medications, antihypertension medications, oral hypoglycemic agents and insulin use. Estimates of KDMAge acceleration and PhenoAge acceleration were demonstrated per SD increase.

### Mediation effect of LV dysfunction on the association between BA and mortality

After adjusting for potential confounders (Supplementary Table 3), there were significant associations between LVMI and *E/e*’ ratio with mortality, suggesting a mediation effect. Table 4 further shows the mediation analysis results of LVMI and *E/e*’ ratio on the relationship between BA acceleration and mortality. The association between BA acceleration and mortality was partly mediated by LVMI and *E/e*’ ratio. Specifically, for KDMAge acceleration, LVMI and *E/e*’ explained 3.96% and 4.17% of the total effect on all-cause mortality, and 4.85% and 5.87% of the total effect on cardiovascular mortality, respectively. For PhenoAge acceleration, the corresponding values were 3.40% and 5.75% for all-cause mortality, and 3.84% and 6.91% for cardiovascular mortality.

**Table 4:** Mediation analysis of the mediation effect of BA acceleration on all-cause mortality and cardiovascular mortality via left ventricular structure and function

		Total effect	Direct effect	Mediation effect		Proportion of mediation (95% CI)
Mediators	Exposures	HR (95% CI)	HR (95% CI)	HR (95% CI)	*P* value	
**All-cause mortality**						
LVMI, g/m^2^	KDMAge acceleration	1.57 (1.41, 1.76)	1.52 (1.36, 1.73)	1.014 (1.005, 1.086)	0.002	3.96% (1.40%, 22.89%)
	PhenoAge acceleration	1.21 (1.05, 1.38)	1.20 (1.02, 1.36)	1.005 (1.001, 1.044)	0.002	3.40% (0.67%, 43.50%)
E/e’ ratio, %	KDMAge acceleration	1.56 (1.41, 1.75)	1.53 (1.38, 1.72)	1.015 (1.004, 1.036)	<0.001	4.17% (1.15%, 10.14%)
	PhenoAge acceleration	1.21 (1.06, 1.39)	1.19 (1.04, 1.37)	1.009 (1.002, 1.025)	0.010	5.75% (1.13%, 21.07%)
**Cardiovascular mortality**						
LVMI, g/m^2^	KDMAge acceleration	1.48 (1.27, 1.74)	1.46 (1.22, 1.69)	1.016 (1.006, 1.103)	0.008	4.85% (1.92%, 31.97%)
	PhenoAge acceleration	1.19 (1.01, 1.43)	1.18 (1.00, 1.41)	1.006 (1.001, 1.048)	0.046	3.84% (0.32%, 71.96%)
E/e’ ratio, %	KDMAge acceleration	1.47 (1.26, 1.73)	1.44 (1.23, 1.69)	1.019 (1.006, 1.045)	0.002	5.87% (2.05%, 15.84%)
	PhenoAge acceleration	1.19 (1.01, 1.42)	1.18 (0.98, 1.40)	1.011 (1.002, 1.029)	0.008	6.91% (-0.6%, 41.33%)

All models were adjusted for sex, age, drinking, smoking, history of hypertension, coronary artery disease, diabetes, atrial fibrillation and stroke, use of lipid lowering medications, antihypertension medications, oral hypoglycemic agents, and insulin.

*LVMI* left ventricular mass index, *RWT* relative wall thickness, *HR* hazard ratio, *CI* confidence interval.

## Discussion

**Fig. 3:**
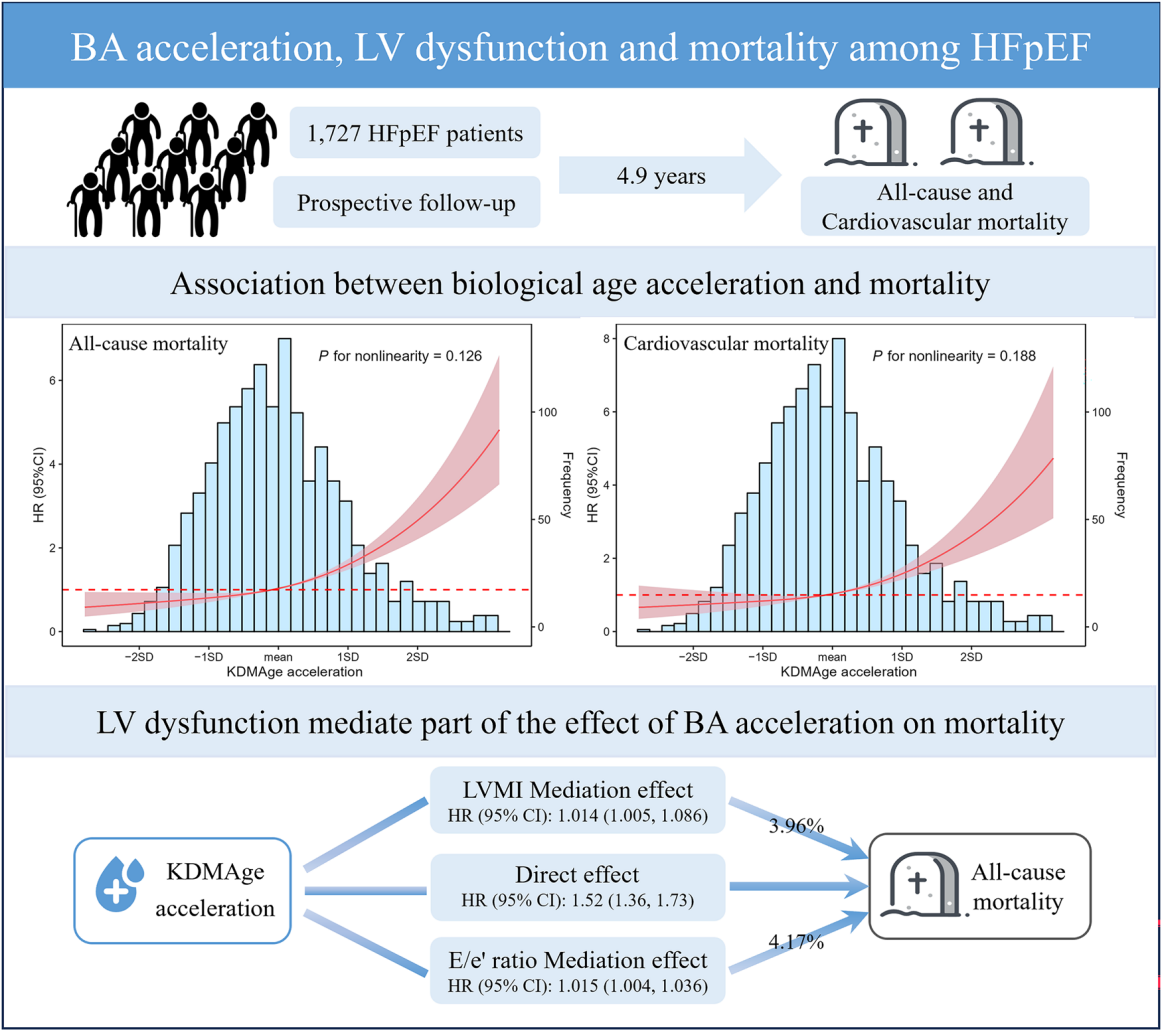
Integrated view of biological aging, cardiac function, and mortality in in patients with heart failure with preserved ejection fraction. BA biological aging, LV left ventricular, LVMI left ventricular mass index.

This study investigated the relationship between biomarker-based BA, cardiac structure, and mortality in individuals with HFpEF (Fig. 3). Our findings revealed that elevated BA was significantly associated with worsening left ventricular dysfunction and an increased risk of both all-cause and cardiovascular mortality in individuals with HFpEF. Furthermore, left ventricular dysfunction partially mediated the association between BA and HF outcomes, with mediation proportions ranging from 3.4% to 6.9%. These results underscore the prognostic value of BA and may indicate a potential pathway through which accelerated biological aging is associated with adverse HF outcomes in Chinese individuals with HFpEF.

Over the past decades, the predictive value of BA has been extensively reported in relation to longevity, hospital admissions, and the incidence of cardiovascular diseases in healthy populations^22–24^. In HF and HF-related outcomes, prior studies predominantly focused on BA measured by leukocyte telomere length^11^. However, the application of epigenetic age and telomere length in large-scale population studies remains challenging due to their high costs and technical complexity^19^. Recently, biomarker-based BA has emerged as a practical and robust measure, showing strong associations with mortality, heart failure incidence, and cardiac morphology and function^19,20,24^. While evidence regarding biomarker-based BA in individuals with HF is limited, it has already demonstrated prognostic utility in chronic kidney disease populations^25^. This highlights a critical research gap in understanding the role of BA, particularly biomarker-based metrics, in HFpEF, including its relationship with HF outcomes and cardiac structure and function.

Consistent with the previous findings in healthy populations and individuals with other chronic diseases^20,25^, the present study demonstrated that advanced biomarker-based BA in HFpEF was associated with an increased risk of all-cause and cardiovascular mortality. Furthermore, in individuals with HFpEF, BA was significantly associated with marked LV hypertrophy and impaired diastolic function, which partially mediated the relationship between BA and mortality.

The mechanisms by which BA predicts mortality, cardiac morphology, and cardiac function likely involve multiple domains. BA incorporates various cardiovascular risk factors, which may partially reflect endothelial dysfunction, inflammation, multiorgan impairment, and cardiometabolic capacity in individuals with HFpEF^16,24^. Genetically, BA metrics are correlated with glycated hemoglobin but capture distinct aspects of aging^26^. KDMAge appears more strongly associated with systolic blood pressure and lipid metabolism pathways, whereas PhenoAge shows stronger correlations with inflammatory and hematological pathways^26^. Similar to the molecular pathways through which chronological age contributes to HF, chronic inflammation, long-term exposure to elevated systolic blood pressure, and glycated hemoglobin may lead to telomere shortening, diastolic dysfunction, and mitochondrial impairment in cardiomyocytes^5,7^.These mechanisms may also explain the stronger predictive value of BA for all-cause mortality in individuals with both diabetes and HFpEF, as their clinical presentation aligns with the definition of diabetic cardiomyopathy, which is partially driven by inflammatory cytokines and mitochondrial dysfunction (e.g., involving SIRT3)^27,28^. Notably, BA was not associated with indicators of LV systolic function, such as LVEF, which differentiates HF subtypes. HFpEF is characterized by increased LV mass and reduced LV cavity size, suggesting that its contribution to mortality is primarily through LV diastolic dysfunction rather than systolic dysfunction^29,30^.

This study highlights the potential clinical value of biomarker-based BA in predicting endpoints in HFpEF and provides exploratory insights into how cardiac morphology and function may be involved in these associations. First, both PhenoAge and KDMAge emerge as effective biomarker-based predictors of aging for HF outcomes in HFpEF, offering a more precise risk stratification tool than chronological age alone. Second, the assessment of cardiac morphology and LV diastolic function is essential in clinical practice, as these parameters may help explain the observed association between BA and mortality. This insight might aid clinicians in stratifying individuals with HFpEF based on LV morphology, LV function, and aging processes. Third, future clinical trials on HFpEF may consider incorporating these factors into subject recruitment and population-specific treatments or exploring the use of BA as a surrogate marker and subclinical outcome for mortality. Finally, these findings contribute exploratory evidence that may inform future research on HFpEF management and encourage investigation into whether targeting biological aging could translate into survival benefits.

The present study has several strengths, including the construction of two biomarker-based BA metrics, the inclusion of individuals with HFpEF, a substantial sample size, and a long follow-up period. However, several limitations should be acknowledged. First, participants were retrospectively recruited from a single tertiary hospital in Southern China and were all hospitalized at least once, which may introduce selection bias and limit generalizability, particularly to asymptomatic individuals. Second, biomarkers were measured only at baseline, precluding the assessment of longitudinal changes in BA over the course of HFpEF progression. Third, our cohort included a high proportion of hypertensive HFpEF patients, which may restrict generalizability to non-hypertensive populations; however, subgroup analyses stratified by hypertension status yield consistent results. Fourth, the KDMAge and PhenoAge algorithms were developed from the U.S. NHANES III cohort and applied without recalibration, which may introduce bias due to population-level differences in biomarker distributions and disease patterns. Although this approach is widely used in non-U.S. cohorts^17,31^, future studies with local reference data are needed to recalibrate and validate these algorithms. Finally, as with other observational studies, reverse causation and residual confounding cannot be fully excluded despite adjustment for major covariates, and the mediation effects of LVMI and E/e′ accounted for only a small proportion of the association between BA acceleration and mortality. Taken together, these findings should be interpreted with caution and warrant validation in prospective, multi-center cohorts before clinical application.

Biomarker-based BA, whether measured by KDMAge or PhenoAge, shows potential predictive value for LV dysfunction, all-cause mortality, and cardiovascular mortality in individuals with HFpEF. Moreover, cardiac morphology and LV diastolic function may help explain the observed associations between BA and HF endpoints. Exploring the prognostic significance of BA acceleration in adverse cardiac remodeling and investigating its potential mechanisms may help inform future strategies for risk stratification and management in HFpEF.

## Methods

### Study design and participants

This retrospective study utilized data from the RED-CARPET study (REal-world Data of CARdiometabolic ProtEcTion), a real-world cohort conducted at the First Affiliated Hospital of Sun Yat-Sen University that collected clinical data from hospitalized patients with cardiometabolic diseases and concurrently performed prospective follow-up for clinical outcomes. The study was registered in the Chinese Clinical Trials Registry (Registration number: ChiCTR2000039901) on November 14, 2020.

In this study, a total of 2428 patients hospitalized for signs or symptoms of congestion and diagnosed with HFpEF were retrospectively enrolled from the RED-CARPET study between June 2016 and July 2021. The diagnostic criteria for HFpEF were based on the European Society of Cardiology’s guidelines for HF^1^, which include (1) typical signs and symptoms of heart failure (e.g., NYHA class II–IV); (2) LVEF ≥ 50%; and (3) evidence of cardiac structural or functional abnormalities on echocardiography, such as (a) left ventricular mass index (LVMI) > 115 g/m² for males or > 95 g/m² for females, (b) relative wall thickness (RWT) > 0.42, (c) *E/e*’ ratio at rest > 9, or (d) N-terminal pro-brain natriuretic peptide (NT-proBNP) > 125 pg/mL for sinus rhythm or >365 pg/mL for atrial fibrillation. Patients lacking BA-related biomarkers or longitudinal follow-up data were excluded. A total of 1727 HFpEF patients were included in the analysis, as shown in Supplementary Fig. 1.

This study adhered to the principles of the Declaration of Helsinki and was approved by the Ethics Review Committee of the First Affiliated Hospital of Sun Yat-Sen University. Written informed consent was obtained from all participants or their representatives prior to study enrollment.

### Data collection and definitions

Baseline data for this study were collected through an electronic clinical management system in a hospital. Demographic characteristics, smoking and drinking history, medical history, and medication history were self-reported by patients. Systolic blood pressure (SBP) and diastolic blood pressure (DBP) were measured by nurses trained in standardized procedures using a sphygmomanometer after the patient rested for 5 minutes during a morning assessment. Body mass index (BMI) was calculated as weight (kg) divided by height squared (m²). Notably, clinical blood markers used to calculate BA—including liver and renal function indexes, lipids, fasting glucose, and blood cell counts—were analyzed using standard techniques on venous blood samples collected after overnight fasting (>8 h). Hypertension was defined as SBP ≥ 140 mm Hg and/or DBP ≥ 90 mm Hg, or the use of antihypertensive medication, or a self-reported history of hypertension. Diabetes was defined as fasting glucose ≥ 7.0 mmol/L, hemoglobin A1c (HbA1c) ≥ 6.5%, the use of medical treatment for diabetes, or a self-reported history of type 2 diabetes mellitus. In addition, we assessed the HF_2_PEF score for each patient^32^, which is calculated as follows: atrial fibrillation contributes 3 points; obesity, defined as a body mass index (BMI) exceeding 30 kg/m², adds 2 points; and each of the following factors—age over 60 years, use of at least two antihypertensive medications, *E/e*′ ratio greater than 9, and pulmonary artery systolic pressure (PASP) above 35 mmHg—adds 1 point.

### Assessment of biological ages

We measured BA using two validated algorithms, KDMAge and PhenoAge, which have been validated in both Chinese^17,33^and European populations^18,34,35^and have demonstrated robust performance in predicting age-related health outcomes. Biomarkers were selected based on their roles in the aging process, prior utilization in research, availability in the data sets, and their statistical significance and correlation strength with CA. For biomarkers that were not normally distributed, logarithmic transformations were applied to approximate a normal distribution. In this study, biomarkers significantly correlated with CA (|r| > 0.1) were included in constructing BA. Ultimately, 10 biomarkers were selected (Supplementary Table 4), including BMI, SBP, DBP, Albumin, the natural log-transformation of blood urea nitrogen (lnBUN), high-density lipoprotein cholesterol (HDL-C), low-density lipoprotein cholesterol (LDL-C), triglycerides, white blood cell (WBC) count, and red blood cell (RBC) count.

The two BA assessment methods, KDMAge and PhenoAge, initially utilized blood chemistry data obtained from the NHANES 1988–1994 (NHANES III) dataset^13^. KDMAge was formulated by performing multiple regression analyses between selected biomarkers and CA within the reference population, which facilitated a quantitative evaluation of systemic integrity decline. In contrast, PhenoAge was created by analyzing various factors related to mortality risk, employing elastic-net Gompertz regression to predict the likelihood of death.

An individual’s KDMAge prediction corresponds to the chronological age at which his/her physiological state is approximately normal. KDMAge is derived from a series of regressions of individual biomarkers against chronological age in a reference population. As previously described, the equation takes information from n regression lines of chronological age against n biomarkers^13^.The formula is as follows:

1 $$\mathrm{KDMAge}=\frac{{\sum }_{j=1}^{n}\left({x}_{{j}}-{q}_{{j}}\right)\left(\frac{{k}_{{j}}}{{s}_{j}^{2}}\right)+\frac{\mathrm{CA}}{{s}_{\mathrm{BA}}^{2}}}{\mathop{\sum }\limits_{j=1}^{n}\left(\frac{{k}_{{j}}}{{s}_{j}^{2}}\right)+\frac{1}{{s}_{\mathrm{BA}}^{2}}}$$

where *x* is the value of biomarker *j* measured for an individual. For each biomarker *j*, the parameters *k*, *q*, and *s* are estimated from a regression of chronological age on the biomarker in the reference sample. *k*, *q*, and *s* are the regression intercept, slope, and root mean squared error, respectively. *S*_BA_ is a scaling factor equal to the square root of the variance in chronological age explained by the biomarker set in the reference sample. CA is chronological age.

The PhenoAge algorithm is based on multivariate analysis of mortality hazards^36^. The original PhenoAge algorithm was developed using elastic-net Gompertz regression of mortality with 42 biomarkers in NHANES III. The formulas are shown in Eqs. (2) and (3):

2 $${\text{PhenoAge}}=141.50225\,+\frac{{\text{ln}}\left(-0.00553\right)\times {\text{ln}}(1-{{\text{MortalityScore}}}_{j})}{0.09165}$$

3 $${{\rm{MortalityScore}}}_{j}={CDF}\left(120,{x}_{j}\right)=1-{e}^{{-e}^{{x}_{j}b}\left(\exp \left(120* \gamma \right)-1\right)/\gamma }$$

where *xb* represents the linear combination of biomarkers from the fitted model, γ is an ancillary parameter estimated from the data, and CDF (120, ${x}_{j}$) denotes the probability that the *j*th individual will die within the next 120 months. The selected traits, algorithms, and corresponding R code are available in the R package ‘BioAge’ at https://github.com/dayoonkwon/BioAge^13^ and in the relevant publications^18,34,35,37,38^.

To quantify differences in BA among patients, BA acceleration was assessed as the residual differences between estimated BA and CA, which indicates whether an individual is biologically older or younger than expected for their chronological age. A positive residual reflects accelerated biological aging (biologically older than chronological age), while a negative residual indicates decelerated aging. To ensure that the effects of the two BA measures (KDMAge and PhenoAge) remained consistent and enable standardized interpretation, BA acceleration was z-score standardized to a mean of 0 and a standard deviation of 1 in continuous analyses.

### Echocardiographic measurements

Comprehensive transthoracic echocardiography was performed using commercially available ultrasound equipment following the American Society of Echocardiography guidelines. For LV structure assessment, LVMI and RWT were measured. LVMI was calculated as LV mass from the Devereux formulation, indexed to body surface area using the Du Bois formula. RWT was calculated as two times the posterior wall thickness divided by the LV internal dimension. LV systolic function was assessed by LV ejection fraction. To assess LV diastolic function, we evaluated early peak diastolic mitral inflow velocity (E), early peak diastolic mitral annular velocity (*e*ʹ) at the septal and lateral wall, and late diastolic velocity (A). iʹ was estimated by dividing E by the mean of lateral and septal *e*ʹ. All echocardiographic images were interpreted by experienced cardiologists who were blinded to the patients’ clinical data, and standardized protocols were strictly followed to ensure consistency and reproducibility.

### All-cause and cardiovascular mortality

The primary outcome was all-cause and cardiovascular mortality, collected by trained staff who contacted patients or their family members by phone, and was further verified through linkage with the National Death Registry Database. Cardiovascular mortality was defined as death related to any cardiovascular event, such as fatal and nonfatal coronary heart disease, stroke, and heart failure^39^.

### Statistical analysis

BA acceleration was categorized into three groups according to tertiles. Continuous variables are presented as mean ± standard deviation (SD) or median with interquartile ranges, and categorical data are presented as numbers and percentages. The Kaplan-Meier method was used to calculate the cumulative incidence of mortality according to the BA acceleration groups, and differences between groups were assessed using the Log-rank test. A Cox proportional hazards regression model was conducted to evaluate hazard ratios (HR) and 95% confidence interval (CI) for the association between BA acceleration and mortality. The association between BA acceleration and echocardiographic measures of LV structure and function was evaluated by linear regression models. Three multivariable models were developed and utilized to adjust for potential confounders of mortality and LV structure and function. Model 1 was adjusted for sex and age at baseline. Model 2 was additionally adjusted for drinking, smoking, history of hypertension, coronary artery disease, diabetes, atrial fibrillation, and stroke. Model 3 was further adjusted for variables in model 2 and lipid-lowering medications, antihypertension medications, oral hypoglycemic agents, and insulin use. A restricted cubic spline regression model with three knots was used to assess the nonlinear dose-response relationship between BA acceleration and mortality. Further subgroup analyses stratified by sex (male or female), age (≤70 or > 70 years), BMI (≤24 or >24 kg/m²), smoking (yes or no), diabetes (yes or no), hypoglycemic agents (yes or no), and and hypertension (yes or no) were employed to examine the consistency of the prognostic impact of BA acceleration on mortality. Additionally, a sensitivity analysis was performed by further adjusting for NT-proBNP to confirm the robustness of the association between BA acceleration and mortality.

To evaluate whether LV structure and function as mediating variables affect the relationship between BA acceleration and mortality among HFpEF patients, we performed a mediation analysis using the CMAverse package^40^ following the approach described by Valeri and Vanderweele^41^. In brief, we assumed the existence of potential interactions between the exposure and the mediator and used regression-based approaches that allowed for the existence of exposure-mediator interaction to estimate the total effect, mediation effect, and direct effect. The mediation effect represented the effect of BA acceleration on mortality that could be explained by its association with the inclusion of the mediators in the model. The direct effect represented the effect of BA acceleration on mortality that was independent of the mediator. The proportion of the association attributable to the mediator (mediation effect/[direct effect + mediation effect]) was estimated to quantify the magnitude of mediation.

All analyses were conducted in R version 4.1.3 (R Foundation for Statistical Computing, Vienna, Austria). A two-sided *P* value < 0.05 was considered statistically significant.

## Supplementary information

Supplementary Information

## Data Availability

The datasets used and/or analyzed during the current study are available from the corresponding author on reasonable request.

## Code availability

The corresponding R code for constructing KDMAge and PhenoAge is available in the R package ‘BioAge’ at https://github.com/dayoonkwon/BioAge.
