# Supplementary material for: Biological aging, left ventricular dysfunction and mortality in patients with heart failure with preserved ejection fraction
Source: NPJ Aging. 2025 Dec 17;11(1):102. doi: 10.1038/s41514-025-00281-6 (PMC12714845; doi:10.1038/s41514-025-00281-6)
Supplement: Supplementary file 1 — Supplementary Information [file 41514_2025_281_MOESM1_ESM.pdf]

Supplementary material

Supplementary Fig.1. Flow chart for selecting HFpEF patients among RED-CARPET study for analysis.

Supplementary Fig.2. Correlation matrix of chronological age, biological ages, and age accelerations.

Supplementary Fig.3. Subgroup analysis of the association between KDMAge acceleration and PhenoAge acceleration with all-cause mortality and cardiovascular mortality.

Supplementary Table 1. Baseline Left ventricular structure and function characteristics of patients.

Supplementary Table 2. Associations of BA acceleration with all-cause mortality and cardiovascular mortality with further adjustment for NT-proBNP.

Supplementary Table 3. Associations of left ventricular structure and function with all-cause mortality and cardiovascular mortality.

Supplementary Table 4. Pearson's correlation coefficients between CA and biomarkers stratified by sex.

Supplementary Fig.1. Flow chart for selecting HFpEF patients among RED-CARPET study for analysis.

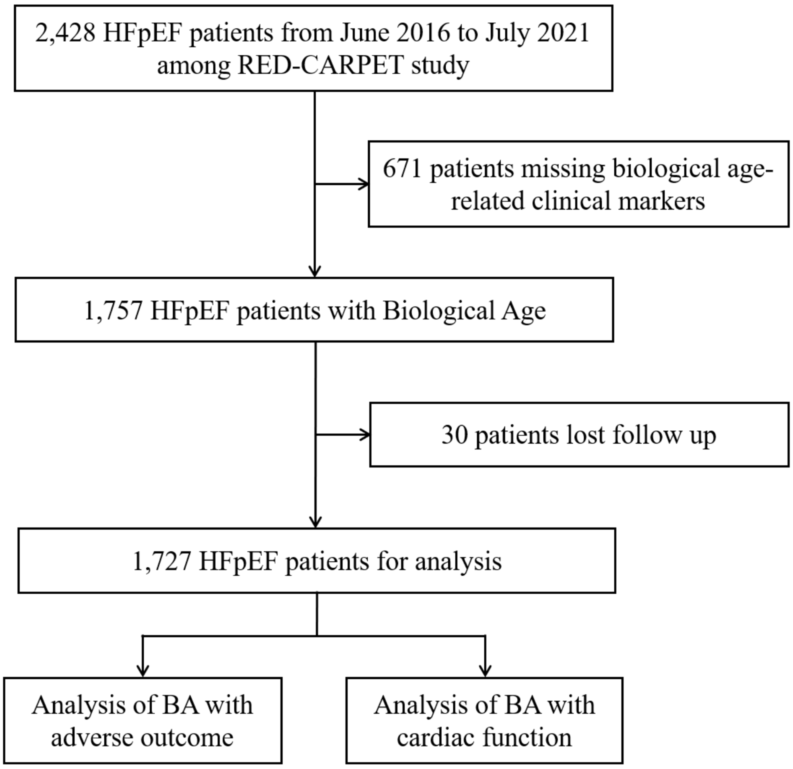

Supplementary Fig.2. Correlation matrix of chronological age, biological ages, and age accelerations.

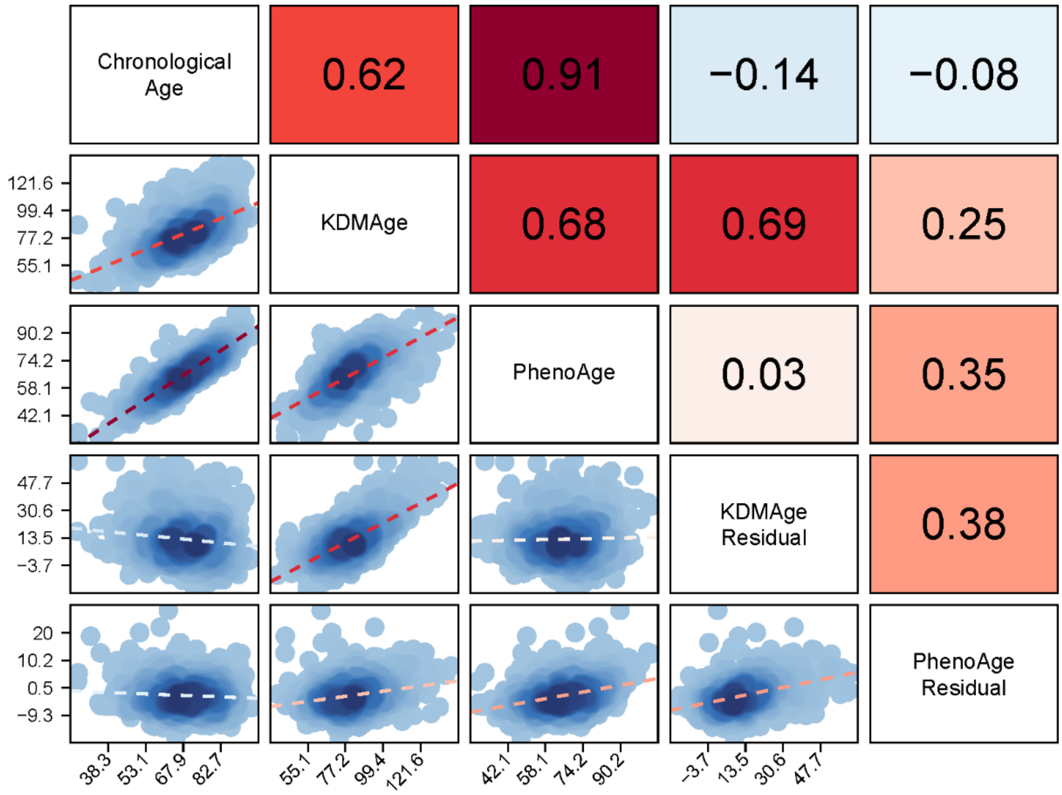

Supplementary Fig.3. Subgroup analysis of the association between KDMAge acceleration and PhenoAge acceleration with all-cause mortality and cardiovascular mortality. **a**, Association between KDMAge acceleration and all-cause mortality. **b**, Association between KDMAge acceleration and cardiovascular mortality. **c**, Association between PhenoAge acceleration and all-cause mortality. **d**, Association between PhenoAge acceleration and cardiovascular mortality. Subgroup analysis included sex (male or female), age (≤ 70 or > 70 years), BMI (≤ 24 or > 24 kg/m^2), smoking (yes or no), diabetes (yes or no) and hypoglycemic agents (yes or no).

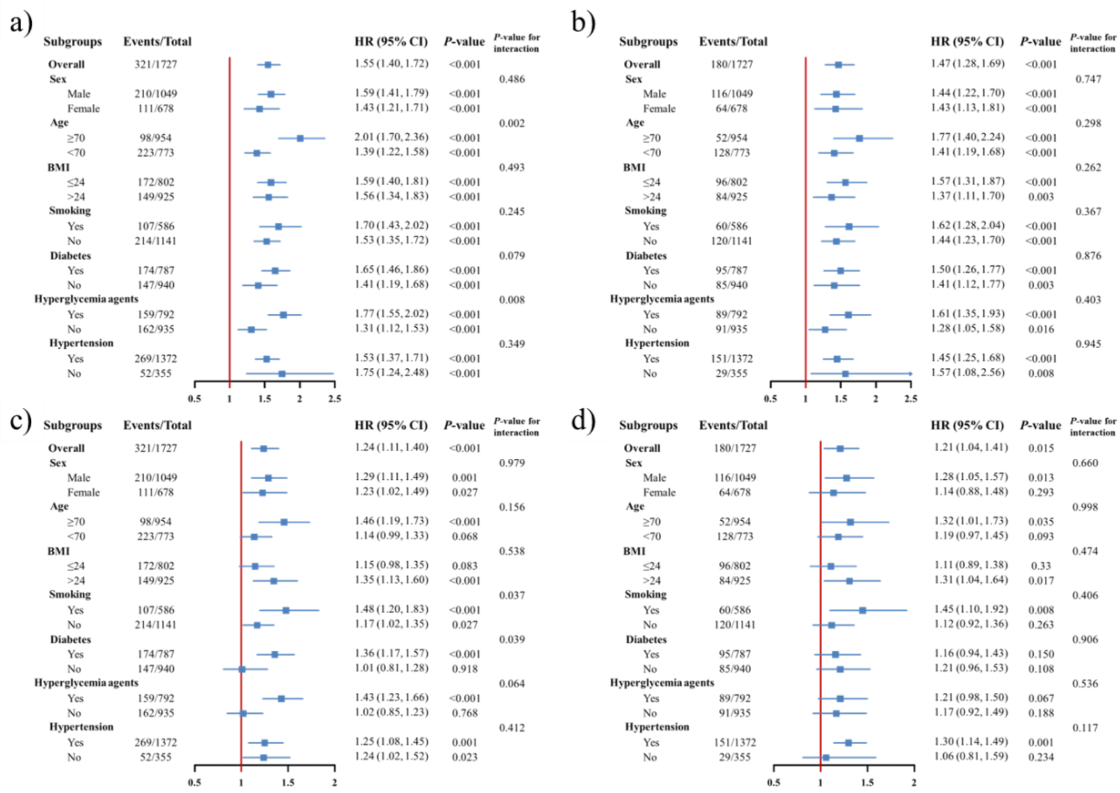

Supplementary Table 1. Baseline Left ventricular structure and function characteristics of patients.

	**Overall (N = 1727)**	**Male (N = 1049)**	**Female (N = 678)**	***P* value**
NYHA functional class				0.596
II	1364 (79.0)	836 (79.7)	528 (77.9)	
III	331 (19.2)	193 (18.4)	138 (20.3)	
IV	32 (1.8)	20 (1.9)	12 (1.8)	
BSA, m^2	1.63 ± 0.18	1.71 ± 0.17	1.52 ± 0.15	<0.001
IVS, mm	11.16 ± 2.11	11.47 ± 2.19	10.69 ± 1.88	<0.001
PWT, mm	9.72 ± 1.75	10.00 ± 1.78	9.28 ± 1.60	<0.001
LVIDd, mm	49.16 ± 6.06	50.37 ± 6.18	47.27 ± 5.36	<0.001
LVESd, mm	30.31 ± 5.44	31.40 ± 5.52	28.63 ± 4.86	<0.001
SV, ml	78.18 ± 23.03	82.19 ± 24.57	71.96 ± 18.81	0.891
LVEF, %	67.73 ± 7.49	66.86 ± 7.60	69.08 ± 7.10	<0.001
Septal e', cm/s	5.99 ± 1.74	6.24 ± 1.76	5.62 ± 1.64	<0.001
Lateral e', cm/s	8.03 ± 2.44	8.35 ± 2.57	7.54 ± 2.13	<0.001
E/e'	11.56 5.18	11.00 ± 5.31	12.43 ± 4.86	<0.001
PASP, mmHg	36.43 ± 10.89	35.90 ± 10.94	37.10 ± 10.81	0.092
LVM, g	198.03 ± 96.43	214.57 ± 113.83	172.55 ± 51.01	<0.001
LVMI, g/m^2	121.09 ± 53.13	125.83 ± 62.60	113.78 ± 32.39	<0.001
RWT	0.40 ± 0.08	0.40 ± 0.09	0.40 ± 0.08	0.128

Data are shown as, n (%), mean ± SD, or median [25th–75th percentile]. Baseline characteristics of the 1,727 eligible HFpEF patients from the RED-CARPET study, stratified by sex. BSA, body surface area; IVS, interventricular septum thickness; PWT, posterior wall thickness; LVIDd, Left Ventricular End-Diastolic Diameter; LVESd, Left Ventricular End-Systolic Diameter; SV, stroke volume; LVEF, left ventricular ejection fraction; PASP, pulmonary artery systolic pressure; LVM, left ventricular mass; RWT, Relative wall thickness.

Supplementary Table 2. Associations of BA acceleration with all-cause mortality and cardiovascular mortality with further adjustment for NT-proBNP.

		Model 1		Model 2	
	**Event/total (%)**	**HR (95% CI)**	***P* value**	**HR (95%CI)**	***P* value**
**KDMAge acceleration**					
**All-cause mortality**					
per SD increase	321/1727 (18.6)	1.62 (1.47, 1.78)	< 0.001	1.47 (1.28, 1.64)	< 0.001
Tertile1	80/575 (13.9)	Reference	-	Reference	-
Tertile2	85/575 (14.8)	1.13 (0.83, 1.55)	0.409	1.08 (0.81, 1.49)	0.488
Tertile3	156/577 (27.0)	2.41 (1.83, 3.19)	< 0.001	2.25 (1.52, 3.01)	< 0.001
P for trend		<0.001		< 0.001	
**Cardiovascular mortality**					
per SD increase	180/1727 (10.4)	1.54 (1.34, 1.77)	< 0.001	1.42 (1.20, 1.67)	< 0.001
Tertile1	48/575 (8.3)	Reference	-	Reference	-
Tertile2	45/575 (7.8)	1.03 (0.67, 1.57)	0.879	1.03 (0.68, 1.60)	0.885
Tertile3	87/577 (15.1)	2.21 (1.52, 3.21)	< 0.001	1.91 (1.21, 2.86)	< 0.001

P for trend		< 0.001		< 0.001	
**PhenoAge acceleration**					
**All-cause mortality**					
per SD increase	321/1727 (18.6)	1.27 (1.13, 1.42)	< 0.001	1.20 (1.07, 1.38)	0.001
Tertile1	85/575 (14.8)	Reference	-	Reference	-
Tertile2	100/575 (17.4)	1.20 (0.89, 1.61)	0.229	1.19 (0.88, 1.62)	0.696
Tertile3	136/577 (23.6)	1.62 (1.22, 2.17)	< 0.001	1.61 (1.19, 2.18)	0.007
P for trend		< 0.001		0.002	
**Cardiovascular mortality**					
per SD increase	180/1727 (10.4)	1.24 (1.06, 1.45)	0.006	1.18 (1.03, 1.37)	0.016
Tertile1	52/575 (9.0)	Reference	-	Reference	-
Tertile2	46/575 (8.0)	0.96 (0.64, 1.46)	0.880	0.92 (0.60, 1.40)	0.985
Tertile3	82/577 (14.2)	1.62 (1.11, 2.38)	0.012	1.60 (1.09, 2.35)	0.012
P for trend		0.005		0.008	

Abbreviations: BA, biological age; HR, hazard ratio; CI, confidence interval; Model 1 Adjusted by sex, age; Model 2 Adjusted by model 1 + drinking, smoking, history of hypertension, coronary artery disease, diabetes, atrial fibrillation and stroke, lipid lowering medications, antihypertension medications, oral hypoglycemic agents, insulin use, and NTproBNP.

Supplementary Table 3. Associations of left ventricular structure and function with all-cause mortality and cardiovascular mortality.

	Model 1		Model 2		Model 3	
	**HR (95% CI)**	***P* value**	**HR (95% CI)**	***P* value**	**HR (95% CI)**	***P* value**
**All-cause mortality**						
LVMI, g/m^2	1.09 (1.05, 1.14)	< 0.001	1.09 (1.04, 1.13)	< 0.001	1.09 (1.04, 1.14)	< 0.001
RWT, cm	1.01 (0.98, 1.13)	0.755	1.01 (0.91, 1.12)	0.823	1.01 (0.90, 1.12)	0.858
E/e' ratio, %	1.20 (1.13, 1.26)	< 0.001	1.21 (1.14, 1.28)	< 0.001	1.16 (1.08, 1.24)	< 0.001
**Cardiovascular mortality**						
LVMI, g/m^2	1.10 (1.04, 1.16)	< 0.001	1.09 (1.03, 1.15)	0.001	1.10 (1.03, 1.16)	0.001
RWT, cm	1.05 (0.91, 1.21)	0.464	1.04 (0.91, 1.19)	0.520	1.04 (0.91, 1.19)	0.525
E/e' ratio, %	1.21 (1.13, 1.30)	< 0.001	1.23 (1.14, 1.32)	< 0.001	1.19 (1.09, 1.29)	< 0.001

Abbreviations: LVMI, left ventricular mass index; RWT, Relative wall thickness; HR, hazard ratio; CI, confidence interval; Model 1 Adjusted by sex, age; Model 2 Adjusted by model 1 + drinking, smoking, history of hypertension, coronary artery disease, diabetes, atrial fibrillation and stroke; Model 3 Adjusted by model 2 + lipid lowering medications, antihypertension medications, oral hypoglycemic agents and insulin use. Estimates of LVMI, RWT and E/e' ratio were demonstrated per SD increase.

Supplementary Table 4. Pearson's correlation coefficients between CA and biomarkers stratified by sex.

	**Total**		**Male**		**Female**	
	r	***P***	***r***	***P***	***r***	***P***
**Physical measurements**						
Height, m	-0.309	< 0.001	-0.225	< 0.001	-0.261	< 0.001
Weight, kg	-0.274	< 0.001	-0.281	< 0.001	-0.093	0.003
BMI, kg/m^2	**-0.107**	< 0.001	-0.203	< 0.001	0.024	0.454
SBP, mm Hg	**0.139**	< 0.001	0.103	< 0.001	0.184	< 0.001
DBP, mm Hg	**-0.177**	< 0.001	-0.200	< 0.001	-0.128	< 0.001
**Blood biomarkers**						
Albumin, g/L	**-0.165**	< 0.001	-0.190	< 0.001	-0.135	< 0.001
Alkaline phosphatase, U/L	0.006	0.786	0.021	0.440	-0.045	0.195
Gamma glutamyl transferase, U/L	-0.048	0.111	-0.024	0.523	-0.059	0.241
Total Bilirubin, mg/dL	-0.086	< 0.001	-0.050	0.068	-0.110	< 0.001
Uric acid	0.035	0.172	0.028	0.395	0.119	0.003
LnCreatine, mg/dL	0.025	0.211	0.007	0.786	0.217	< 0.001
LnBUN, mg/dL	**0.134**	< 0.001	0.083	0.001	0.267	< 0.001
Total cholesterol, mg/dL	-0.088	< 0.001	-0.122	< 0.001	-0.113	< 0.001
HDL-C, mg/dL	**0.161**	< 0.001	0.126	< 0.001	0.102	< 0.001
LDL-C, mg/dL	**-0.114**	< 0.001	-0.138	< 0.001	-0.129	< 0.001
Triglyceride, mg/dL	**-0.177**	< 0.001	-0.218	< 0.001	-0.163	< 0.001
Glucose, mmol/L	-0.023	0.241	-0.038	0.142	-0.028	0.382
HbA1c, %	0.005	0.795	-0.009	0.733	-0.026	0.458
WBC count	**-0.148**	< 0.001	-0.172	< 0.001	-0.092	0.006
RBC count	**-0.249**	< 0.001	-0.254	< 0.001	-0.149	< 0.001
Mean cell volume, fL	0.070	0.00112	0.099	< 0.001	0.041	0.238

Abbreviations: CA, chronological age; BMI, body mass index; SBP, systolic blood pressure; DBP, diastolic blood pressure; WBC, white blood cell; RBC = red blood cell; BUN, blood urea nitrogen; HDL-C, high-density lipoprotein cholesterol; LDL-C, low-density lipoprotein cholesterol.
